# A Case Control Study Reveals that Polyomaviruria Is Significantly Associated with Interstitial Cystitis and Vesical Ulceration

**DOI:** 10.1371/journal.pone.0137310

**Published:** 2015-09-01

**Authors:** Benjamin J. Winter, Helen E. O'Connell, Scott Bowden, Marcus Carey, Damon P. Eisen

**Affiliations:** 1 Faculty of Medicine, Dentistry and Health Sciences, University of Melbourne, Parkville, Victoria, Australia; 2 Department of Surgery, Royal Melbourne Hospital, Parkville, Victoria, Australia; 3 Victorian Infectious Diseases Reference Laboratory, Wreckyn St, North Melbourne, Victoria, Australia; 4 Department of Urogynaecology, Royal Women’s Hospital, Flemington Rd, Parkville, Victoria, Australia; 5 Victorian Infectious Diseases Service at the Doherty Institute, Royal Melbourne Hospital, Melbourne, Victoria, Australia; 6 Department of Medicine, Royal Melbourne Hospital, University of Melbourne, Parkville, Victoria, Australia; Oklahoma University Health Sciences Center, UNITED STATES

## Abstract

**Objectives:**

To investigate whether polyomaviruses contribute to interstitial cystitis pathogenesis.

**Subjects and Methods:**

A prospective study was performed with 50 interstitial cystitis cases compared with 50 age-matched, disease-free controls for the frequency of polyomaviruria. Associations between polyomaviruria and disease characteristics were analysed in cases. Polyomavirus in urine and bladder tissue was detected with species (JC virus vs. BK virus) specific, real-time PCR.

**Results:**

Case patients were reflective of interstitial cystitis epidemiology with age range from 26–88 years (median 58) and female predominance (41/50 F). There was a significant increase in the frequency of polyomavirus shedding between cases and controls (p<0.02). Polyomavirus shedding, in particular BK viruria, was associated with vesical ulceration, a marker of disease severity, among interstitial cystitis cases after adjustment for age and sex (OR 6.8, 95% CI 1.89–24.4). There was a significant association among cases between the presence of BK viruria and response to intravesical Clorpactin therapy (OR 4.50, 95% CI 1.17–17.4).

**Conclusion:**

The presence of polyomaviruria was found to be associated with the ulcerative form of interstitial cystitis. Clorpactin, which has anti-DNA virus activity, was more likely to improve symptoms in the presence of BK viruria. These data from this pilot study suggest associations between polyomaviruria and interstitial cystitis warranting further investigation.

## Introduction

Searching for new insights into pathogenesis and potential treatments for interstitial cystitis is important given the high prevalence and substantial morbidity of the condition. [1] While interstitial cystitis is currently defined as occurring only in the absence of infection [2], a search for pathogens with potential associations with painful bladder syndrome continues, driven by a perceived need to expand potential therapeutic targets for this disabling condition. This applies to polyomaviruses that, after establishing latent infection in the urothelium during childhood, are frequently shed in the urine. [3] The role of BK virus (BKV), one of the five polyomaviruses, in causing urinary pathology rather than just urinary shedding is established. Where immunosuppressive regimens are used to prevent organ transplant rejection BKV causes renal graft nephropathy [4] and haemorrhagic cystitis [5], particularly in haematological stem cell transplants. JC virus (JCV) is even more prevalent [6] and is shed asymptomatically more commonly in the urine than BKV in healthy individuals. [7] To date it has not been linked with urinary pathology but rather, causes the cerebral white matter disease, progressive multifocal leukoencephalopathy.

A recent, small, case-control study [8], has shown a trend to increased BKV viruria in IC patients. In the absence of definitive evidence regarding these pathogens we have taken a systematic, prospective approach to the study of a possible pathogenic role for polyomaviruses in interstitial cystitis. We have used a case-control study to investigate patients with cystoscopic and clinical features of interstitial cystitis compared with patients without features of interstitial cystitis.

## Subjects and Methods

### Ethics statement

The project titled ‘Investigating the role of the polyomaviruses BKV and JCV in causing Interstitial Cystitis’ was approved by the Royal Melbourne Hospital Human Research Ethics Committee and the Royal Women’s Hospital Human Research Ethics Committee; project number 2009/018 and 10/55 respectively. Written consent was obtained from all Interstitial Cystitis patients and controls prior to enrolment in this study.

### Patient and control characteristics

Patients with interstitial cystitis were identified from the Royal Melbourne Hospital Private Medical Centre, and Royal Women’s Hospital in Melbourne, Australia. In order to be eligible for this study, patients required a previous diagnosis of interstitial cystitis (the presence of either glomerulations or ulceration on cystoscopy and hydrodistension) and a history consistent with the Interstitial Cystitis Database Study eligibility criteria. [9]

Other patients with painful bladder syndrome who responded to oral medications, physical therapy or bladder retraining who had never progressed to cystoscopy and hydrodistension were excluded.

Patient records were reviewed and relevant information from the cystoscopy and hydrodistension that diagnosed interstitial cystitis was recorded. Assessment of the severity of current interstitial cystitis symptoms was performed using the validated O’Leary-Sant symptom and problem indices and cystoscopic findings. [10]

An age-matched cohort of controls who were selected for the absence of cystitis due to any cause was recruited to compare the frequency of polyomaviruria with interstitial cystitis patients. Selection of controls was consistent with the epidemiological study investigating the prevalence of polyomaviruria performed by Zhong et al., [7] in which participants were grouped by age into decades and not separated by gender.

### Polyomavirus detection

Upon recruitment into the study, patients with interstitial cystitis and controls were screened for the presence of urinary polyomavirus by PCR. Polyomavirus PCR testing was performed with BKV and JCV DNA being quantified using a real-time 5’ nuclease PCR assay which employed a probe and primers that anneal specifically to the coding region of the respective large T antigen.

The presence of polyomavirus in bladder biopsy specimens was sought by immunohistochemistry in archived biopsy material from patients with interstitial cystitis. These tissue samples were stained with immunoperoxidase to detect the presence of polyomavirus replication, using a monoclonal antibody against the simian virus 40 (SV40) T antigen and a polyclonal antibody against capsid proteins SV40, which both bind human BKV and JCV. Bound test antibodies were detected via the streptavidin-biotin method and were revealed using diaminobenzidine as a chromogen.

### Sample size calculation and statistical method

BKV is present in the urine in up to 30% of 50–59 year old individuals and JCV in 60% of the same population. [7] A, priori, we based sample size calculations on the assumption that BKV would be present in 30% more interstitial cystitis patients than the general population (i.e. BKV in 60% of interstitial cystitis patients and, using the same odds ratio, JCV in 84% of interstitial cystitis patients). Under this assumption, a sample of 50 pairs of interstitial cystitis patients and non-cystitis controls would be sufficient to detect a difference of this magnitude in the frequency of both viruses.

The case control study used χ^2^ testing for comparison of the BKV and JCV prevalence binary variable in age-matched case—control pairs. Two-sample T-testing was used to compare the levels of BKV and JCV between interstitial cystitis patients and controls. Binary logistic regression was used to compare the clinical characteristics of interstitial cystitis patients with and without polyomaviruria. The characteristics analysed were ulceration as seen on cystoscopy, and a response to intravesical Clorpactin defined as reduction in pain, urinary frequency and nocturia as determined during outpatient follow-up. Intravesical treatment with Clorpactin is not offered at Royal Women’s Hospital, therefore these patients were not included in the analysis of a response to Clorpactin.

### Cystoscopic treatment

Prior to treatment with cystoscopy, hydrodistension and intravesical Clorpactin, all patients were refractory to alternative therapy including amitriptyline, bladder retraining and pelvic muscle therapy, non-steroidal anti-inflammatory agent, pentosan polysulphate and DMSO intravesical instillations. Cystoscopy and hydrodistension was performed in a standardized fashion. [11]

## Results

A total of 107 patients with previously diagnosed interstitial cystitis were invited to participate in the study. Forty-four of these either declined to participate in the study, or were unable to be contacted. Eight patients who returned the consent form were subsequently deemed ineligible as they no longer satisfied the ICDB criteria, either due to a change in diagnosis, bladder removal or resolution of urinary symptoms. Five patients were lost to follow-up and therefore unable to provide a urine specimen for testing.

Fifty patients with interstitial cystitis and fifty age-matched controls without cystitis were investigated for polyomaviruria. Case control demographics are demonstrated in Table 1, with no significant difference in age-matching of cases and controls (p = 0.82).

**Table 1:** Case control analysis.

	IC cases	Controls	
**Age range**	26–88	20–87	
**Mean age**	57.44	56.66	
**Median age**	58.5	58.5	
**Sex—female n (%)**	41 (82)	27 (54)	0.69
**Polyomaviruria n (%)**	28 (56)	16 (32)	0.02
**Mean BKV urinary load log**_**10**_	2.92	1.84	0.09
**Mean JCV urinary load log**_**10**_	3.29	1.74	0.02

Interstitial cystitis cases and disease free control demographics and associations with polyomaviruria.

Polyomavirus urinary shedding was more prevalent in patients with interstitial cystitis compared to age-matched controls. (p<0.02). Table 1. Urinary viral loads were log_10_-transformed for statistical analysis. There was a significant difference in the mean levels of JC virus urinary shedding in patients with interstitial cystitis compared to controls. (p<0.02). A trend to higher levels of BKV was also present.

Cystoscopic and hydrodistension data were available for 49/50 patients with interstitial cystitis, 27 of whom had ulceration. Significant associations were present between urinary shedding of both polyomaviruses and ulceration; (BKV: OR 6.80, 95% CI 1.89–24.2 and JCV: 4.29, 1.29–14.26). Polyomavirus urinary shedding was also positively associated with ulceration at cystoscopy. Twenty of the 23 patients treated with Clorpactin demonstrated symptomatic improvement during outpatient review post cystoscopy. A beneficial response to intravesical Clorpactin was correlated specifically with BKV urinary shedding (OR = 4.50, 95% CI 1.17–17.4). A non-significant trend to Clorpactin response and JCV polyomaviruria was also present (2.79, 0.77–10). Of the 20 patients reporting benefit following Clorpactin, 14 of these had the ulcerative form of interstitial cystitis. The three patients who did not respond to Clorpactin all had the non-ulcerative form of interstitial cystitis. O’Leary-Sant questionnaires were available for 43/50 patients, with a mean score of 17.6 and range 3–34. No association existed between O’Leary-Sant symptom severity and polyomavirus urinary shedding (p = 0.78).

Thirteen interstitial cystitis patients with polyomaviruria had archived bladder tissue biopsy examined for evidence of polyomaviruses. Six interstitial cystitis patients without polyomaviruria also had their bladder tissue biopsy examined. Bladder biopsy samples analysed were taken up to ten years ago. PCR testing of bladder tissue biopsies of these 19 patients yielded one positive result for polyomavirus DNA. A weakly positive level of JCV was detected in an interstitial cystitis patient with polyomavirus viruria. This 81-year-old female had BKV and JCV present in her urine, both at a viral load of 10^7^ copies per mL. This female had the presence of ulceration and glomerulations on cystoscopy, a response to treatment with Clorpactin and a severity score of 10; all factors that indicate that her IC was severe. The biopsy was taken in August 2001. Polyomavirus immunohistochemistry tests performed on the 19 bladder tissue biopsy samples were all negative.

## Discussion

Possible links between interstitial cystitis causation and infection have been sought repeatedly to date. Bacterial pathogens that frequently cause urinary tract infection like *Escherichia coli* and cell-wall deficient organisms such as *Mycoplasma hominis* were studied, among others, showing no associations. [12] Similarly, viruses that establish latent or persistent infection like herpes simplex, adenovirus, cytomegalovirus, and human papillomavirus were absent from the bladder biopsies of interstitial cystitis patients. [13] Polyomavirus detection was not included in the study methodology.

Our study builds on previous attempts to that have shown a possible contribution of polyomavirus infection to the pathogenesis of IC. [14] The recent study of patients with IC compared with controls with non-cystitis associated urological conditions showed a non-significant trend to higher incidence of BKV shedding in IC patients with ulceration. [8]

We have previously reported a single case of interstitial cystitis occurring in a diabetic man who was successfully treated with intravesical cidofovir. A substantial, 5-log10, reduction in the urinary PCR levels of polyomaviruses was seen with intravesical cidofovir, which coincided with a sustained reduction in symptoms of painful bladder syndrome. [15] This individual had been refractory to a full spectrum of therapeutic approaches and symptoms of his IC were extremely severe.

In this pilot study we have demonstrated a number of significant associations between interstitial cystitis and polyomaviruses warranting further investigation of the relationship. There was a significant increase in polyomaviral shedding when interstitial cystitis cases were compared with age matched disease free controls. The ulcerative form of the disease, typically occurring in patients with lower volume under anaesthetic was significantly associated with the presence of polyomavirus. Whether this finding represents cause or effect is uncertain as vesical ulceration may predispose to greater shedding of latent virus from the denuded urothelium.

Despite no infection being found as a cause of interstitial cystitis to date, the search for infective precipitants for some patients with interstitial cystitis is still important as new treatments may then be considered for this disabling condition. We believe that polyomaviruses are possible contributors to the pathogenesis of interstitial cystitis. These viruses occur commonly in the population with seroprevalence of approximately 80% and 40–60% for BKV and JCV respectively. [6] The fact that polyomaviruses establish latency in the urothelium with frequent, asymptomatic shedding [7], distinguishes them from other viruses examined previously in interstitial cystitis patients. The pathogenicity of BKV in the urogenital tract is established. In the setting of haematopoietic stem cell transplantation, severe BKV haemorrhagic cystitis may be lethal. [16]

Our study provides us with evidence of polyomaviruses’ association with IC compared with a cohort of unselected controls with no symptoms of cystitis. It may possibly be instructive to perform a validation study with a control group consisting of patients with other forms of cystitis such as recurrent bacterial infection. As we have suggested above, it may be that where the bladder is inflamed, polyomavirus shedding may be more prevalent therefore reducing or negating the effect size we have seen in this study.

Polyomaviruses were only detected in one of the interstitial cystitis patients’ bladder biopsy specimens. The biopsies were typically obtained from the posterior wall of the bladder consistent with common Urological practice. Cystoscopic biopsies are not taken from ulcerative areas because of the risk of bladder perforation. This low yield for polyomavirus detection in the bladder tissue may be due to sampling error, the archival nature of the specimens, shedding from the upper urinary tract or it may be negative evidence for a causative association between interstitial cystitis and polyoma virus. Future studies could include biopsies in the vicinity of the ulcerative area with small cold cup biopsies.

Therapeutic options for interstitial cystitis related to polyomaviruria may be opened up if this association is borne out by further studies. Fluoroquinolones like ciprofloxacin [17], and other agents, both antiviral (cidofovir) [18] and immunomodulatory (leflunamide) [19] have all been shown to have anti-polyomavirus activity in vitro. Of particular relevance to existing treatments used in interstitial cystitis, Clorpactin has been shown to have in vitro antiviral activity against DNA viruses including vaccinia and herpes simplex along with the RNA virus HIV. [20] Clorpactin has not been tested yet for polyomavirus activity and this along with Pentosan polysulfate sodium (Elimiron), and iAluril, should be investigated for anti-polyomavirus activity. In patients with low capacity (typically ulcerative) interstitial cystitis, many of whom are considered for radical therapy such as urinary diversion, we would recommend studies to determine if polyomaviruria is present before such radical measures and consideration of further therapy with Clorpactin.

Reports of therapeutic use of lower concentrations of Clorpactin suggest that there may be opportunities to systematically compare this with other agents possessing anti-viral or other therapeutic effect. Compelling patient testimonials who had only cystectomy and diversion as an option when they used Clorpactin led to its more widespread use when other treatments had failed. We are aware that there has been a medical trend against this agent though it is possible it may have been an innocent bystander in the reported cases. [21] The small risk of ureteral fibrosis should be mitigated against by prospective detection of vesico-ureteral reflux cystogram prior to its use. In cases where reflux was present, ureteral occlusion balloons were used. In future our use of Clorpactin is likely to be in the context of clinical research. We have had no complications with its use and repeated use has been associated with bladder preservation rather than progressive loss of bladder volume associated with repeated bladder cautery.

Potentially higher levels of endogenous steroids due to chronic pain could influence the frequency of polyomaviruria in cases with interstitial cystitis. None of the fifty cases with interstitial cystitis were taking exogenous steroids, however the effect of chronic pain on endogenous steroid levels was not studied. This weakness could be addressed by including a control group of patients with chronic pain syndromes to test for polyomavirus frequency. Furthermore, ACTH, CRF, sympathetic nervous system responses and diurnal salivary cortisol levels could be studied.

A weakness of this study is that the data used for assessing a response to Clorpactin was retrospective. Patient records were reviewed, and information was obtained from the follow-up appointment three months post treatment. The treating clinician determined a response to Clorpactin as symptom reduction, improvement in voided volumes and reduced nocturia for longer than 3 months. Hydrodistension alone has been demonstrated to have symptomatic benefit for a few weeks [22], therefore symptomatic reduction in the manner of 2–4 weeks was not considered significant, and these patients were classified as non-responders for the purpose of the study. There were twenty responders and 3 non-responders to Clorpactin, all of which returned the questionnaire. O’Leary-Sant questionnaires were conducted post-treatment to investigate a relationship between polyomavirus shedding and symptom severity. Prospective analysis of a response to Clorpactin and novel agents such as iAluRil would allow the use of pre and post-treatment urinary testing and O’Leary-Sant questionnaires. Another weakness was that recent treatment for interstitial cystitis was not analysed prior to the collection of the questionnaires, which could potentially bias symptom severity and urinary polyomavirus shedding.

Interstitial cystitis is a common and disabling condition. The antecedent injuries and abnormalities that lead to the endpoint of a denuded urothelial glyclosaminoglycan layer may be contributed to either primarily by polyomavirus shedding. Alternately, patients with interstitial cystitis may develop ulceration due to polyomavirus shedding. In either scenario, polyomaviruria in interstitial cystitis may identify a group of these patients who could benefit from targeted anti-infective therapy, particularly with Clorpactin.

## Supporting Information

S1 Table Case control analysis of polyomaviruria. (XLSX)

S2 Table Interstitial cystitis disease and treatment associations. (XLSX)
